# Assessment of transvalvular flow jet angle in aortic dilation patients using 4D flow jet shear layer detection method

**DOI:** 10.1186/1532-429X-16-S1-P47

**Published:** 2014-01-16

**Authors:** Julio Garcia, Michael Markl, Pim van Ooij, Susanne Schnell, Jeremy D Collins, SC Malaisrie, James C Carr, Alex J Barker

**Affiliations:** 1 Radiology, Northwestern University, Chicago, Illinois, USA; 2 Biomedical Engineering, Northwestern University, Chicago, Illinois, USA; 3 Division of Cardiothoracic Surgery, Northwestern University, Chicago, Illinois, USA

## Background

Patients with aortic dilation often present an eccentric transvalvular flow jet. The angle of the flow jet from the aorta centerline, or the flow jet angle (FJA), has been reported as a risk factor in bicuspid aortic valve patients [1]. In recent studies we introduced a the jet shear layer detection (JSLD) method for the automated characterization of transvalvular flow structure across the aortic valve [2,3]. The objective of this study was to evaluate FJA in patients with aortic dilation using the 3D JSLD structure obtained from 4D flow MRI.

## Methods

**Figure 1:**
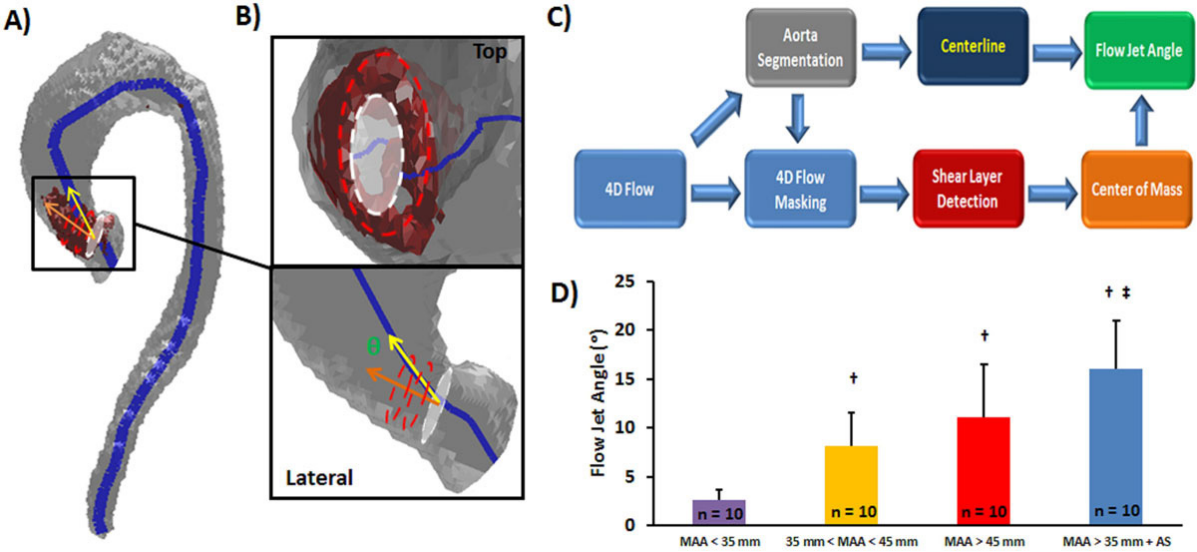
**Flow jet angle estimation using 4D flow**. Panel A shows the aorta segmentation obtained from 4D flow angiography. Aorta segmentation (gray) was used to compute volume centerline (blue line). Jet shear layer detection (JSLD) was computed from 4D flow data, 3D JSLD structure (red), corresponding to vena contracta region, was obtained by normalizing JSLD. White line: aortic valve location; Red lines: sections of 3D JSLD structure; Yellow arrow: centerline vector; Orange line: 3D JSLD center of mass vector. Panel B shows a view from the top of the aortic valve at vena contracta region, a lateral view of vena contracta region with schematic lines for aortic valve location, 3D JSLD structure, centerline vector and 3D JSLD center of mass vector. Panel C shows the workflow for computing transvalvular flow jet angle from 4D flow data. Panel D shows a comparison of patient's groups. †: significant difference with MAA<35 mm; ‡: significant difference with 35 mm<MAA<45 mm; MAA: Mid ascending aorta diameter.

This study included 40 patients with aortic dilation and aortic tricuspid valves participants (age = 58 ± 16 years, female = 11, aortic stenosis = 10). Mid-ascending aorta (MAA) diameter and transvalvular peak velocity (Vpeak) were used to assess aortic dilation, aortic stenosis severity (AS, Vpeak>2 m/s), and classify patients into four groups: Group 1 (MAA<35 mm); Group 2 (35 mm<MAA<45 mm); Group 3 (MAA>35 mm); Group 4 (MAA>35 mm and AS). 4D flow MRI was performed at 1.5T and 3T systems with full thoracic aorta volume coverage in a sagittal oblique slab (spatial resolution = 2.5 × 2.1 × 3.2 mm 3; temporal resolution = 40-50 ms). 4D flow data were used to compute a PC-MRA image and aorta volume segmentation was performed using Mimics (Materialise, Leuven, Belgium). The isolated aorta segmentation was used to automatically compute the vessel centerline, to mask 4D flow data, and compute 3D JSLD structure using Matlab (Natick, MA, USA). FJA workflow is summarized on Figure 1.

## Results

Patient characteristics and measurements are presented in Table 1. A significant difference between groups was observed for age (p < 0.05), Vpeak (p < 0.001) and FJA (p < 0.001) using an ANOVA test. Group 2-4 were older than Group 1. Group 4 presented higher Vpeak in comparison with Group 1-3 due to AS. When comparing FJA for defined group's population, significant differences were found between Group 2-4 vs. Group 1 (p < 0.05) and Group 4 vs. Group 2 (p < 0.05), see Figure 1D. Higher FJA was found in Group 3 and Group 4. Interestingly, the Pearson's correlation coefficient between Vpeak and FJA was r = 0.54, p < 0.01, and between ejection fraction and FJA was r = 0.38, p < 0.05.

**Table 1:** Subject Characteristics

	All	MAA < 35 mm	35 mm < MAA < 45 mm	MAA > 45 mm	MAA > 35 mm + AS	p-value ANOVA
**n**	**40**	**10**	**10**	**10**	**10**	

Age (years)	58 ± 16	42 ± 17	63 ± 11	62 ± 10	63 ± 14	<0.05

Female (n)	11	2	4	4	1	NS

Ejection Fraction (%)	59 ± 7	57 ± 6	59 ± 8	64 ± 4	60 ± 6	NS

Stroke Volume (mL)	91 ± 30	84 ± 15	84 ± 21	100 ± 47	96 ± 30	NS

Sinus of Valsalva Diameter (mm)	41 ± 5	39 ± 8	41 ± 3	42 ± 5	39 ± 4	NS

Mid Ascending Aorta Diameter (mm)	39 ± 7	30 ± 4	41 ± 3	47 ± 2	42 ± 3	<0.001

Peak Velocity (m/s)	1.6 ± 0.9	1.1 ± 0.4	1.3 ± 0.3	1.4 ± 0.16	2.9 ± 0.9	<0.001

Flow Jet Angle(degrees)	9 ± 6	3 ± 1	8 ± 3	11 ± 5	16 ± 5	<0.001

MAA: Mid Ascending Aorta; AS: Aortic Stenosis

## Conclusions

The assessment of FJA can be automated using the volumetric information of 3D JSLD structure which relates the 3D JSLD structure to the aorta centerline, as obtained from 4D flow data. FJA was significantly higher in patients with severe aortic dilation and concomitant AS. Future longitudinal studies are needed to evaluate the impact of FJA in aortic dilation severity and altered flow patterns.

## Funding

Grant support by NIH R01HL115828, NUCATS Dixon Award, AHA 13SDG14360004. CONACyT postdoctoral fellow grant (223355).
